# Health-related quality of life in children, adolescents and young adults with self-harm or suicidality: A systematic review

**DOI:** 10.1177/00048674231165477

**Published:** 2023-04-10

**Authors:** Ngoc Le, Yared Belete Belay, Long Khanh-Dao Le, Jane Pirkis, Cathrine Mihalopoulos

**Affiliations:** 1 Deakin Health Economics, Deakin University, Burwood, VIC, Australia; 2 Monash University Health Economics Group (MUHEG), School of Public Health and Preventive Medicine, Monash University, Melbourne, VIC, Australia; 3 School of Pharmacy, Mekelle University, Mekelle, Ethiopia; 4 Centre for Mental Health, Melbourne School of Population and Global Health, University of Melbourne, Parkville, VIC, Australia

**Keywords:** Health-related quality of life, systematic review, self-harm, suicidality, young people

## Abstract

**Objective:**

Self-harm and suicidality are associated with substantial social and economic burden, especially among children, adolescents and young adults. The aim of this review was to systematically synthesize the literature on the association between health-related quality of life and self-harm/suicidality in children, adolescents and young adults.

**Methods:**

Searches were conducted via MEDLINE, PsycINFO, CINAHL, EconLit and EMBASE. Search terms were the combination of the following blocks: (1) self-harm/suicidality, (2) health-related quality of life/well-being/life satisfaction and (3) children/adolescents/young adults. The quality of studies was assessed using the Effective Public Health Practice Project tool.

**Results:**

We identified 23 relevant studies. Findings showed that participants who reported self-harm had lower well-being, life satisfaction or overall health-related quality of life compared to those without self-harm. There was also evidence supporting the association between health-related quality of life and suicidal attempt. However, the results for the association with suicidal ideation remained inconsistent. Additionally, mental health, emotional well-being, physical health, oral health, existential well-being and family quality of life were found to be significant domains associated with self-harm or suicidality. Regarding the quality of included studies, 35% (*n* = 8), 39% (*n* = 9) and 26% (*n* = 6) of studies were scored as ‘Strong’, ‘Moderate’ and ‘Weak’, respectively.

**Conclusion:**

Findings from the review showed that health-related quality of life varied according to the severity of suicidality (from ideation to attempt). There was also no evidence to infer the direction of causality between health-related quality of life and self-harm/suicidality. The findings suggest a need for further research, in particular longitudinal studies to fill identified gaps in the literature.

## Introduction

Self-harm and suicidality are of significant public health concern and pose a substantial social and economic burden, especially among children, adolescents and young adults.^1^ According to international statistics, 13.7% of children and adolescents reported self-harm in their lifetime (Kim-San et al., 2019). Suicide is the leading cause of death among Australians aged 15–24 years and the fourth leading cause of mortality worldwide for this age group (Australian Institute of Health and Welfare [AIHW], 2021; World Health Organization [WHO], 2021).

Self-harm is defined as any behaviour that an individual has deliberately engaged in to cause pain or injury to self, with or without the intent to die (Angelotta, 2015; Lifeline, 2021). These behaviours may include poisoning, cutting, burning, hitting, biting or scraping skin (Lifeline, 2021). Additionally, the term ‘suicidality’ used for the purpose of this review refers to suicidal thoughts and behaviours. Terms such as suicidal ideation, suicidal threat, suicidal plan, suicidal attempt and suicide are all contained in the overarching construct of suicidality. Suicidal behaviours and self-harm are associated with transdiagnostic difficulties as such behaviours depend on many differential factors like intent, the behaviour’s frequency, physical damage level and psychological suffering level (Walsh, 2006). Therefore, self-harm and suicidal behaviours are often used interchangeably although they are not synonymous (Shaw, 2002). This review encompasses both forms of self-harm regardless of suicidal intentions and suicidality.

Health-related quality of life (HRQoL) is a multi-dimensional concept that extends to the physical, mental and social aspects of well-being and functioning (Bullinger, 2002) and can be an important indicator of burden of disease, particularly when comparing the HRQoL of people with the health condition of interest to those without the health condition. There are currently two main categories of HRQoL measurement: *generic instruments* and *disease-specific instruments* (Guyatt et al., 1993). Generic HRQoL instruments are commonly used and comprise a descriptive system consisting of questions assessing different domains of quality of life (QoL). These instruments have the overall advantage that they can be used for broad comparison across populations and to gauge the relative impact of various health care programmes. *Disease-specific instruments* are designed to capture the impact on HRQoL by focusing on a specific area of interest and are therefore theoretically more clinically sensitive to smaller changes and more responsive (Guyatt et al., 1993; Hays, 2005). Among both generic and disease-specific instruments, preference-based instruments incorporate individuals’ preferences for specific health states in a separate scoring algorithm and are used in cost-utility analyses to determine quality-adjusted life years (QALYs) (Guyatt et al., 1993).

Exploring the association between HRQoL and self-harm/suicidality in young people is important from a descriptive, disease burden perspective as well as from an economic perspective. By investigating distinct QoL dimensions, insights into the decrements of a young person’s functioning and well-being may be gained. The research findings will correspondingly help inform understandings of self-harm and suicidal risk, contributing to the development of effective suicide prevention strategies among young populations. The importance of prevention programmes is undeniable in terms of the obvious benefits with regard to lives saved and emotional trauma avoided (Kennelly, 2007). From the perspective of economics, successful suicide prevention interventions also bring other benefits to society in the form of what a person is able to contribute when he or she is saved from suicide (Kennelly, 2007). This is especially crucial as young people are the foundation of future social and economic growth in any society. The impacts of a young person’s self-harm/suicidality on families and community should not be overlooked either. Research has shown that parents who have children with self-harm issues reported intense negative emotions and experienced parenting burden and stress, which resulted in decreased psychological well-being and functioning (Townsend et al., 2021). Family members are extremely affected in cases when a young person dies by suicide. They often blame themselves and are overwhelmed with grief and despair (Cerel et al., 2008; Linn-Gust, 2010). Notably, for every youth suicide death, there are 100–200 suicidal attempts and thousands of young people reporting serious suicidal ideation (HealthDirect, 2021; Miller and Eckert, 2009). For all these reasons, the important role of research on HRQoL in the context of suicide prevention in young people requires further expansion. The aim of this review was to systematically synthesize the literature on the association between HRQoL and self-harm/suicidality in young people. We also explored the evidence of association between preference and non-preference-based QoL and self-harm/suicidality.

## Methods

This review followed the guidelines in the Preferred Reporting Items for Systematic Reviews and Meta-Analyses: the PRISMA statement (Page et al., 2021). The review’s protocol was registered on PROSPERO (CRD42021262734).

### Search strategy

Searches were conducted in the following databases: MEDLINE, PsycINFO, CINAHL, EconLit and EMBASE in July 2021. A supplementary search was also undertaken by hand to identify additional articles. The search terms included a broad range of terms and were combined in the following blocks: (1) self-harm or suicidality, (2) HRQoL or well-being or life satisfaction and (3) children or adolescents or young adults (see Supplemental Appendix 1). Finally, a secondary search was conducted before final analysis to retrieve additional articles for inclusion up to May 2022.

### Inclusion criteria

There was no restriction on publication time for included articles. Only articles in English and articles published in peer-reviewed journals were included. Articles were included in the review if they were quantitative research studies from primary data and met the following criteria.

#### Population

Young people who were under 25 years of age (<25 years) with self-harm/suicidality. Moreover, studies on young people with self-harm/suicidality were eligible for inclusion regardless of whether they had other health conditions.

#### Study designs

There was no restriction on the types of study design (e.g. cohort study, randomized controlled trial or case control study).

#### Reported outcomes

Studies that reported the association between HRQoL/domains of HRQoL and self-harm/suicidality in either direction (either the impact of self-harm/suicidality on HRQoL or the impact of HRQoL on self-harm/suicidality) were included. It should be noted that reported outcomes could be HRQoL scores measured by any non-preference-based or preference-based instruments (with the preference-based scores of such studies usually referred to as utilities). Furthermore, although some researchers suggest that there are major differences among *QoL, well-being* and *life satisfaction*, these terms are often used interchangeably to refer to a person’s subjective perception of aspects of life (CDC, 2018a, 2018b). Therefore, articles that reported outcomes on *well-being* and *life satisfaction* were also considered to be relevant to our research aims and included in the review.

### Study selection and extraction

Data from the searches were imported into an electronic software package (Endnote® version 20) (The EndNote Team, 2013) by the first author (N.L.) with duplicates subsequently removed. All citations were then imported into the Covidence platform. Duplicate removal was also conducted a second time in Covidence. Next, title and abstract screening was conducted by two authors who independently applied the selection criteria to identify relevant studies (N.L., Y.B.B.). Discrepancies in title and abstract screening were resolved based on the consensus of a third author (L.K.-D.L.). Similarly, full text screening was independently conducted by the same two authors (N.L., Y.B.B.), and discrepancies were resolved by the third author (L.K.-D.L.). Data extraction was conducted by the primary author (N.L.) with double checking by another author (Y.B.B.). Data were extracted into an Excel file.

### Quality assessment

Using the Effective Public Health Practice Project (EPHPP, 2022) tool, the quality of included studies was evaluated. The tool comprises six components: (1) selection bias; (2) study design; (3) confounders; (4) blinding; (5) data collection methods; and (6) withdrawals and dropouts. Each component was assessed and rated as ‘Strong’, ‘Moderate’ or ‘Weak’. Based on each component rating, the overall study was then rated as ‘Strong’, ‘Moderate’ or ‘Weak’ following the tool’s guidelines. Additionally, the EPHPP tool also includes two components (intervention integrity and analyses) which were assessed but not rated and do not affect the overall study rating. EPHPP is considered to have a very strong methodological rating and its components are universally relevant to many quantitative studies on any health topic (EPHPP, 2022). The tool was reported to have reliability (kappa statistic ranges from 0.61 and 0.74) and have content and construct validity (Thomas et al., 2004). Furthermore, it has been found that the EPHPP quality assessment tool had fair and excellent inter-rater agreement for individual domains and for the final grade (Armijo-Olivo et al., 2012). Therefore, even though EPHPP is not the perfect assessment tool for all studies, it was deemed to be the most appropriate tool to evaluate the quality of included studies in the review based on their diversity. Quality assessment of included studies in the review was independently conducted by two authors (N.L., Y.B.B.) and discrepancies were identified and resolved through deliberation within the research team.

#### Data synthesis

We used narrative synthesis to report findings from the included studies. Due to the data’s heterogeneity (studied populations with various age groups, various scales/instruments with different specific domains, inadequate information for effect size calculation), a meta-analysis could not be conducted. (Table 1 represents details of specific domains measured within each scale/instrument in the included studies and Table 2 includes descriptions and findings of all studies.)

**Table 1.** Details of instruments used in included studies.

Name of instrument	Studies that used the instrument	Domains measured
*Well-being/Life satisfaction Scales*		
The Personal Well-being Index – School Children (PWI-SC)	Díez-Gómez et al. (2020); Fonseca-Pedrero et al. (2018)	Eight domains: ‘Life as a whole’; Standard of living, Health, Life achievements, Relationships, Safety, Community connectedness and Future security (responses are from 0 to 10, total scores range from 0 to 70)
The Ryff’s Psychological Wellbeing (PWB) Scale	Owusu-Ansah et al. (2020)	Psychological well-being (5-point Likert scale responses, scores range from 18 to 90)
The Spiritual Well-Being Scale (SWBS)	Taliaferro et al. (2009)	Religious well-being (RWB), Existential well-being (EWB) (6-point scale responses, total scores range from 20 to 120 with scores range of subscales are from 10 to 60)
The Satisfaction with Life Scale (SWLS)	Parker et al. (2018); Whitlock et al. (2015)	Life satisfaction (7-point scale responses, total scores range from 5 to 35)
Warwick-Edinburgh Mental Wellbeing Scale (WEMWBS)	Morey et al. (2017); Quarshie et al. (2021)	Mental well-being (5-point Likert scale responses, scores range from 14 to 70)
WHO Well-Being Index (WHO-5)	Sisask et al. (2008)	Overall well-being (6-point scale responses, total scores range from 0 to 25)
The Youth Self-Report Scale	Rönkä et al. (2013)	Life satisfaction (response was from 1 to 5, total score was from 1 to 5)
*Generic non-preference-based HRQoL instruments*		
The 1997 Centers for Disease Control and Prevention’s (CDC) Youth Risk Behaviour Survey (YRBS) – modified version	Thatcher et al. (2002)	Six domains: Satisfaction with Self, School, Where they live, Friendships, Family, and Overall life (7-point Likert scale responses, total scores range from 6 to 42)
Brief Multidimensional Students’ Life Satisfaction Scale-College (BMSLSS-C) and The CDC HRQOL Core Scale	Zullig (2016)	BMSLSS-C includes eight domains: Global QoL, Family, Friends, School, Self, Living, Environment, Romantic relationships, Physical appearance (7-point scale responses, total scores range from 8 to 56) The CDC HRQOL Core Scale includes four questions: self-perceived health (5-point scale response), physical health, mental health, global measure of both physical and mental health (7-point scale days with 1 = 0 day to 7 = 30 days)
The Child Perception Questionnaire (CPQ 11–14) – short version	Al-Bitar et al. (2022)	Four domains of OHRQoL: oral symptoms, functional limitations, emotional well-being and social well-being (The total CPQ scores ranged from 0 to 59)
The Chinese Six-Item QOL questionnaire	Wang et al. (2019)	Six domains: Physical health, Psychological health, Economic conditions, Study, Family relationship, and Relationships with non-family associates (5-point scale responses, total scores range from 6 to 30)
Combination of instruments: The Chinese Emotional Intelligence Scale (C-EIS-R); the Social Problem-Solving Inventory (C-SPSI-R); the Chinese Hopelessness Scale (C-HOPE); Parent–Adolescent Communication: FACS and MACS; the Chinese Family Assessment Instrument (C-FAI)	Kwok and Shek (2010)	Personal QoL (Emotional Competence [5-point scale responses, scores range from 12 to 60]; Social Problem-Solving [5-point scale response, scores range from 25 to 125]; Hopelessness [4-point scale response, scores range from 10 to 40]); Family QoL (Parent–Adolescent Communication [no information on scores range]; Family Functioning [5-point scale response])
KIDSCREEN	Resch et al. (2008)	Total HRQoL (total scores range from 0 to 100)
Inventory of Life Quality for Children and Adolescents (ILK)	Balazs et al. (2018); Gyori et al. (2021)	One global scale and six different domains: School, Family, Peer relations, Being alone, Somatic health, and Mental state (each scale ranges from 1 to 5)
The Questionnaire for Measuring Health-Related Quality of Life in Children and Adolescents–Revised (KINDL-R)	Algorta et al. (2011)	Six subscales of QoL: Physical, Emotional, Self-esteem, Family, Friends and School, with higher scores indicating better QoL (5-point scale responses, total scores range from 0 to 24)
Taiwanese QOL Questionnaire for Adolescents	Lee et al. (2017)	3 subscales Pain-related QoL (each subscale ranges from 0 to 100)
The 36-Item Short Form (SF-36)	Ballester et al. (2021); Luo et al. (2021)	General health, role-physical, role-emotional, mental health, social functioning, vitality, physical functioning and bodily pain (each scale ranges from 0 to 100)
*Preference-based instruments*		
Child Health Utility 9D (CHU9D)	Le et al. (2021)	Nine health dimensions: Worry, Sadness, Pain, Tiredness, Annoyance, School work/homework, Sleep, Daily routine, Ability to join in activities. Utility scores range from 0 to 1

OHRQoL: oral-health-related quality of life; FACS: father–adolescent communication scale; MACS: mother–adolescent communication scale; KIDSCREEN: a health-related Quality of Life Questionnaire for Children and Adolescents.

**Table 2.** Included studies of HRQoL in young people with self-harm/suicidality.

ID	Study	Population	Study design	HRQoL instrument used	Self-harm/suicidality definition/instrument used	Rater	Main findings
*Studies using Well-being/Life satisfaction Scales*							
1	Díez-Gómez et al. (2020)	*n* = 1506 adolescent students, including 667 males (44.3%) Aged 14–19 years	Cross-sectional study	Personal Well-Being Index – School Children (PWI-SC)	The Paykel Suicide Scale (PSS)	Self-reported	The subgroup with suicidal ideation and ‘high suicide risk’ (or suicidal attempt) showed lower scores on subjective well-being compared to the non-suicide risk subgroups (*p* < 0.001)
2	Fonseca-Pedrero et al. (2018)	*n* = 1664 participants, including 782 males (47%) Aged 14–19 years	Cross-sectional study	Personal Well-Being Index – School Children (PWI-SC)	Paykel Suicide Scale (PSS)	Self-reported	Participants who reported suicidal ideation showed lower emotional well-being and life satisfaction compared to the non-suicidal ideation group (*p* < 0.001, *d* = 0.47 moderate effect size)
3	Morey et al. (2017)	*n* = 2000 adolescents Aged 13–18 years	Cross-sectional study	Warwick-Edinburgh Mental Wellbeing Scale (WEMWBS)	Child and Adolescent Self-Harm in Europe (CASE)	Self-reported	Self-harm was associated with a significantly lower well-being score
4	Owusu-Ansah et al. (2020)	*n* = 1003 students Mean age = 20.53 years (SD = 5.95) Female 49.5%	Cross-sectional study	The Ryff’s Psychological Wellbeing (PWB) Scale	Suicide-related questions	Self-reported	Only suicidal attempt but not suicidal ideation was significantly predicted by subjective or psychological well-being (*p* = 0.007)
5	Parker et al. (2018)	*n* = 1579 Year 11 and 12 students Female 50.2%	Cross-sectional study	The Satisfaction With Life Scale (SWLS)	Self-developed questions	Self-reported	Adolescents who had experienced suicidality has lower scores on the SWLS than those without suicidality (*p* < 0.05, *d* = 0.8 large effect size)
6	Quarshie et al. (2021)	*n* = 450 school-going deaf adolescents Aged 12–14 years Female 38%	Cross-sectional study	The 14-item Warwick-Edinburgh Mental Well-Being Scale (WEMWBS)	The 2012 Ghana WHO-Global School-Based Health Survey (WHO-GSHS)	Self-reported	High subjective mental well-being was associated with reduced odds of both suicidal ideation and attempt (*p* < 0.001)
7	Rönkä et al. (2013)	*n* = 7014 Age < 16 years Female (51.9%)	Cohort study	The Youth Self-Report Scale	The Youth Self-Report Scale	Self-reported	Those who were dissatisfied with life (girls: OR 3.3; boys: OR 3.3) were more likely to report DSH
8	Sisask et al. (2008)	*n* = 178 suicidal attempters Age 15–24 years Female 65.7%	Cross-sectional study	WHO Well-Being Index (WHO-5)	Pierce Suicidal Intent Scale (PSIS)–revised version	Self-reported	Low level of well-being was associated with high level of suicidal intent (*p* < 0.05)
9	Taliaferro et al. (2009)	*n* = 457 Age 18–24 years Female 74%	Cross-sectional study	The Spiritual Well-Being Scale (SWBS)	The 25-Item Adult Suicidal Ideation Questionnaire (ASIQ)	Self-reported	‘Higher religious, existential, and total spiritual well-being were associated with lower levels of suicidal ideation (*p* < 0.01)’
10	Whitlock et al. (2015)	*n* = 836 students with history of repeated NSSI Mean age 21.3 years Female 78.3%	Cross-sectional study	The McMaster Family Assessment Device	The Non-suicidal Self-Injury Assessment Tool	Self-reported	Individuals who had stopped NSSI reported greater life satisfaction (AOR = 1.22, 95% CI, [1.07, 1.40])
*Studies using generic non-preference-based HRQoL instruments*							
11	Al-Bitar et al. (2022)	699 school children (aged 13–14 years) 48.5% females	Cross-sectional study	The Child Perception Questionnaire (CPQ 11–14)	The Child and Adolescent Self-harm in Europe (CASE) questionnaire	Self-reported	The participants who reported self-harm scored higher total CPQ scores, CPQ individual dimension scores (or lower OHRQoL) than the participants who reported no self-harm (*p* < 0.001)
12	Algorta et al. (2011)	*n* = 138 youth with paediatric bipolar disorder Age 5–18 years 82% female, 18% male	Cross-sectional study	The Questionnaire for Measuring Health-Related Quality of Life in Children and Adolescents–Revised (KINDL-R)	KSADS interview	Self-reported and parent proxy report	Family QoL (*p* < 0.005, *d* = 1.03 large effect size) and Total QoL (*p* < 0.05, *d* = 0.4 medium effect size) scales were significantly worse in the Suicidal Attempt group => result only found for Suicidal Attempt group The Total QoL was no longer significantly different after controlling for Family QoL
13	Balazs et al. (2018)	*n* = 134 Age 13–18 years Female 46.3%	Cross-sectional study	Inventory of Life Quality for Children and Adolescents (ILK)	MINI KID 2.0	Self-reported	Adolescents showing medium/high level of suicidal risk showed higher scores on ILK self-report (or lower QoL) than adolescents with no/low level of suicidal risk (*p* < 0.001, *d* = 1.7 large effect size)
14	Ballester et al. (2021)	1560 young adults aged between 18 and 24 (56.5% were females)	Prospective cohort study	The 36-Item Short Form (SF-36)	Mini-International Neuropsychiatric Interview (MINI)	Self-reported	Poor quality of life was significantly a risk factor for the incidence of suicide
15	Gyori et al. (2021)	*n* = 191 adolescents Age 13–18 years Girls 49.7%	Cross-sectional study	Inventory of Life Quality for Children and Adolescents (ILK)	The Deliberate Self-Harm Inventory (DSHI)	Self-reported and parent proxy report	Adolescents engaged in NSSI were rated significantly higher (meaning worse QoL) in physical health, mental health (*p* < 0.01), family and global well-being (*p* < 0.001) There is no significant difference between self and parent QoL ratings among adolescents in the NSSI group
16	Kwok and Shek (2010)	*n* = 5557 students Age 11–18 years Female 46.9%	Cross-sectional study	Combination of: the Chinese Emotional Intelligence Scale (C-EIS-R); the Social Problem-Solving Inventory (C-SPSI-R); the Chinese Hopelessness Scale (C-HOPE); Parent–Adolescent Communication: FACS and MACS; the Chinese Family Assessment Instrument (C-FAI)	The 13-item Suicidal Ideation Sub-Scale (C-SIS)	Self-reported	Suicidal ideation was negatively related to family functioning (*p* < 0.0017)
17	Lee et al. (2017)	*n* = 6150 students Age 11–18 years Female 52.3%	Cross-sectional study	Taiwanese QOL Questionnaire for Adolescents	The 5-item questionnaire derived from the Kiddie Schedule for Affective Disorders and Schizophrenia	Self-reported	Increased risks of suicidality were significantly associated with a low level of satisfaction with pain-related QOL (*p* < 0.001)
18	Luo et al. (2021)	9057 medical students and included 579 medical students with migraine (77% females); mean age of 19.6 ± 1.6 years	Cross-sectional study	The 36-Item Short Form (SF-36)	Self-Rating Idea of Suicide Scale (SIOSS)	Self-reported	High quality of life was significantly a protective role against suicidal ideation
19	Resch et al. (2008)	*n* = 2863 Aged 7–17 years	Cross-sectional study	KIDSCREEN	The child behaviour check list (CBCL) and the youth self-report (YSR)	Self-reported and parent proxy report	Children and adolescents exhibiting suicidal behaviour reported significantly lower HRQoL (*p* < 0.001, *d* = 0.89–1.05 large effect sizes). Parents rated their children better than the children scored themselves on almost all scales
20	Thatcher et al. (2002)	*n* = 4565 public high school students Female 53.8%	Cross-sectional study	The 1997 Centers for Disease Control and Prevention’s (CDC) Youth Risk Behaviour Survey (YRBS)–modified version	The 1997 Centers for Disease Control and Prevention’s (CDC) Youth Risk Behaviour Survey (YRBS)–modified version	Self-reported	For White females, attempted suicide was significantly associated with satisfaction with family, school and self (*p* < 0.001). For both White and Black males, attempted suicide was significantly associated with satisfaction with overall life (*p* < 0.001)
21	Wang et al. (2019)	*n* = 26,688 students Aged 15–20 years Female 51.3%	Cross-sectional study	The Chinese Six-Item QOL questionnaire	Item 15 of the Symptom Checklist-90-R	Self-reported	After controlling for demographic, lifestyle and clinical covariates, a high QOL score (means poorer QoL) (OR = 1.09, *p* < 0.001) remained significantly associated with Suicidal Ideation (*d* = 0.96 large effect size)
22	Zullig (2016)	*n* = 723 students Aged 18–24 years Female 54%	Cross-sectional study	Brief Multidimensional Students’ Life Satisfaction Scale-College (BMSLSS-C) and The CDC HRQOL Core Scale	Question: ‘Approximately how often, if at all, in the past six months have you intentionally cut, burned, punched, bit, or scratched yourself or otherwise hurt yourself physically without the intention to kill yourself?’	Self-reported	DSH is a significant predictor for all life satisfaction domains, overall life satisfaction (*p* < 0.001). Surprisingly, DSH was only weakly associated with satisfaction with family. Effect sizes ranged from 0.42 (Living Environment) to 1.18 (Overall), indicating medium to large effects
*Studies using preference-based instruments*							
23	Le (2021)	*n* = 2967 adolescents Aged 11–17 years Female 48.5%	Cross-sectional study	Child Health Utility 9D (CHU9D)	A question regarding deliberate harm or injury, with or without suicidal intent was asked	Self-reported	Adolescents who self-harmed, had suicidal ideation had significantly lower utilities scores compared to those who did not have such conditions (*d* = 1.38–1.39, *p* < 0.001 with moderate to large effect sizes)

OR: odds ratio; AOR: adjusted odds ratio; CI: confidence interval; OHRQoL: oral-health-related quality of life; DSH: deliberate self-harm; NSSI: nonsuicidal self-Injury; KSADS: kiddie schedule for affective disorders and schizophrenia; FACS: father–adolescent communication scale; MACS: mother–adolescent communication scale; KIDSCREEN: a health-related quality of life questionnaire for children and adolescents.

## Results

**Figure 1.**
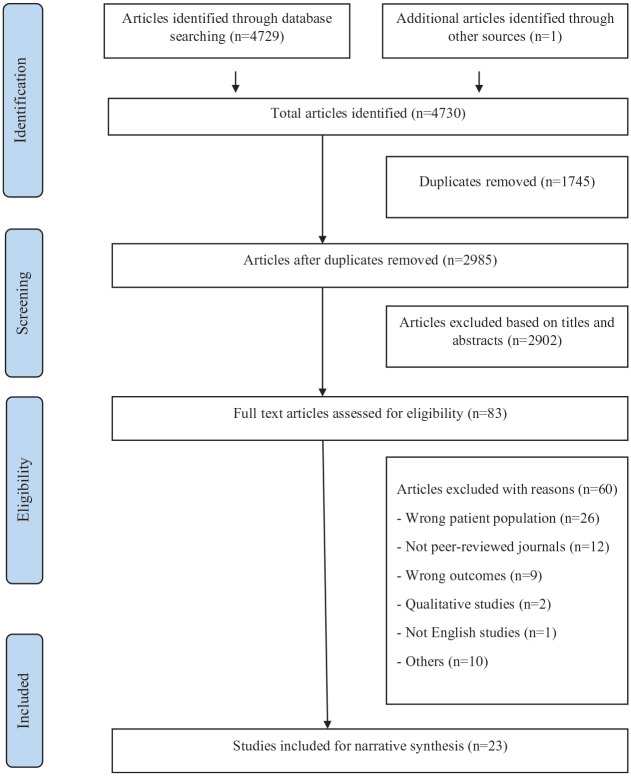
PRISMA flow diagram (Page et al., 2021).

A total of 4730 articles were identified from all databases. After removing duplicates and screening, 23 studies were included in the final synthesis. Details are shown in the PRISMA flowchart (Figure 1).

### Study characteristics

In summary, among the 23 included studies, there were 20 studies based on self-report and 3 studies based on both self-report and parent proxy report (Algorta et al., 2011; Gyori et al., 2021; Resch et al., 2008). Sample sizes ranged from 134 (Balazs et al., 2018) to 26,688 (Wang et al., 2019). Two studies focused on children and adolescents aged 5–18 years (Algorta et al., 2011; Resch et al., 2008); 14 studies focused on adolescents aged 11–20 years; and 7 studies focused on young adults aged 18–24 years. Notably, all studies were cross-sectional, and none used a longitudinal design. There were five studies investigating self-harm/suicidality in young people in clinical samples (Algorta et al., 2011; Balazs et al., 2018; Gyori et al., 2021; Quarshie et al., 2021; Sisask et al., 2008) and the remainders were in nonclinical samples. Moreover, there were 6 studies on people with self-harm and 17 studies on people with suicidality. Most studies were conducted in Europe (*n* = 8), followed by the United States (*n* = 5), Asia (*n* = 5), Australia (*n* = 2), Africa (*n* = 2) and South America (*n* = 1).

### Quality assessment of included studies

Based on the EPHPP tool, 35% (*n* = 8), 39% (*n* = 9) and 26% (*n* = 6) of studies were scored as ‘Strong’, ‘Moderate’ and ‘Weak’, respectively. Almost all studies used reliable and valid data collection tools (Cronbach’s alpha ranged from 0.6 to 0.9) and were therefore rated as ‘Strong’ for this component. For example, two studies used the Satisfaction with Life Scale (SWLS) with Cronbach’s alpha = 0.74 (Parker et al., 2018; Whitlock et al., 2015); two studies used the 36-Item Short Form (SF-36) with Cronbach’s alpha > 0.8 (Ballester et al., 2021; Luo et al., 2021). Additionally, most studies did not describe or did not blind both the outcome assessors and participants and were therefore rated as ‘Moderate’ for Blinding component. Details of included studies and their quality assessment are shown in Supplemental Appendix 2.

### Measures

A wide variety of instruments was used to measure HRQoL, well-being or life satisfaction across included studies. Ten studies used well-being or life satisfaction scales, of which the Personal Wellbeing Index–School Children (PWI-SC), the SWLS and the Warwick-Edinburgh Mental Wellbeing Scale (WEMWBS) were the most commonly used. Twelve studies used generic non-preference-based HRQoL instruments and only one study used a preference-based HRQoL instrument (the Child Health Utility 9D or CHU9D). Among non-preference-based instruments, the Inventory of Life Quality for Children and Adolescents (ILK) and the SF-36 survey were most frequently used.

### Association between HRQoL and self-harm/suicidality

Among the group of 10 studies using well-being or life satisfaction scales, 3 studies reported results for self-harm and 7 studies reported results for suicidality. Findings generally showed that self-harm was inversely associated with overall well-being or life satisfaction (Morey et al., 2017; Rönkä et al., 2013; Whitlock et al., 2015). In seven studies that reported results for suicidality, there was evidence of different associations formed with well-being or life satisfaction. Specifically, suicidal attempt was significantly related to lower overall well-being, lower mental well-being and life satisfaction (Díez-Gómez et al., 2020; Owusu-Ansah et al., 2020; Parker et al., 2018; Quarshie et al., 2021). The results for suicidal ideation were inconsistent. While four studies found an association between suicidal ideation and lower overall well-being and life satisfaction (Díez-Gómez et al., 2020; Fonseca-Pedrero et al., 2018; Parker et al., 2018; Sisask et al., 2008), one study found no association between suicidal ideation and overall well-being (Owusu-Ansah et al., 2020). It is noteworthy that Owusu-Ansah et al.’s study was overall rated as ‘Weak’, with ‘Weak’ ratings for its study design and confounder component. Furthermore, the subscales/domains of well-being measures such as lower emotional well-being, mental well-being and existential well-being domains were found to be related to suicidal ideation (Fonseca-Pedrero et al., 2018; Quarshie et al., 2021; Taliaferro et al., 2009).

Findings with respect to non-preference-based HRQoL instruments showed that self-harm was significantly associated with worse overall HRQoL (Gyori et al., 2021; Zullig, 2016). There was also evidence that participants who reported self-harm had lower oral-health-related quality of life (OHRQoL), lower physical health and mental health QoL domains than those who did not self-harm (Al-Bitar et al., 2022; Gyori et al., 2021). Notably, two studies both reported the outcome that the family domain was significantly associated with self-harm as measured by the ILK and the Brief Multidimensional Students’ Life Satisfaction Scale-College (BMSLSS-C) instruments (Gyori et al., 2021; Zullig, 2016). It is noteworthy from Zullig’s study, which involved people aged over 18, that self-harm was only weakly associated with the family domain. Additionally, there was a group of nine studies that reported results for suicidality. Findings from five studies showed that suicidal attempt was significantly related to lower overall HRQoL (Algorta et al., 2011; Balazs et al., 2018; Ballester et al., 2021; Lee et al., 2017; Resch et al., 2008). Moreover, suicidal attempt was significantly related to family QoL domains, which were measured by the Questionnaire for Measuring Health-Related Quality of Life in Children and Adolescents–Revised (KINDL-R) and the 1997 Centers for Disease Control and Prevention’s (CDC) Youth Risk Behaviour Survey (YRBS) (Algorta et al., 2011; Thatcher et al., 2002). Both studies involved children and adolescents aged below 18 years. Algorta et al.’s study and Thatcher et al.’s study were rated as ‘Moderate’ and ‘Strong’, respectively. It should be noted that results on suicidal ideation were also inconsistent. While three studies showed evidence of association between suicidal ideation and overall QoL as well as family QoL (Kwok and Shek, 2010; Luo et al., 2021; Wang et al., 2019), the study by Algorta and colleagues found no association between suicidal ideation and QoL when both family QoL and total QoL were in the same model (Algorta et al., 2011).

The only study to find that participants who self-harmed and reported suicidal ideation had significantly lower utility scores than those who did not have such behaviours as measured by the CHU9D instrument was by Le et al. (2021). The quality of this study was rated as ‘Strong’.

It is noteworthy that there were three studies investigating the association between HRQoL and self-harm/suicidality in relation to a mediational relationship with other interested factors. Specifically, Balazs et al. (2018) suggested that HRQoL mediated the relationship between emotional and peer problems and suicidal risk. On the contrary, two other studies reported that the relationship between HRQoL and self-harm/suicidality was mediated by mental disorders or HRQoL affected self-harm/suicidality mainly through hopelessness (Gyori et al., 2021; Kwok and Shek, 2010).

Finally, among the three studies which included both self-report and parent proxy report, two studies discussed differences between self and parent QoL ratings (Gyori et al., 2021; Resch et al., 2008). While Gyori et al. discovered no significant difference between self-rating and parent rating in the nonsuicidal self-injury group (Corrected *p*-value = 0.074), Resch et al. found that parents rated their children/adolescents with higher HRQoL than the children/adolescents scored themselves (*p*-value < 0.001) (Gyori et al., 2021; Resch et al., 2008). Both these two studies were rated as ‘Strong’ for quality assessment.

## Discussion

To the best of our knowledge, this is the first systematic review on the association between self-harm/suicidality and HRQoL in young people. Not surprisingly, our synthesis showed that findings from included studies reported an inverse association between self-harm/suicidality and overall HRQoL. This outcome remained the same irrespective of the types of scales used. The outcome might support the notion that people with relatively poorer HRQoL might be more likely to self-harm or to be suicidal. Alternatively, it might suggest that people who reported self-harm or suicidal individuals might ultimately have poorer HRQoL. Moreover, findings on the association between specific HRQoL domains and self-harm/suicidality provided insights into which aspects of people HRQoL are associated with self-harm/suicidality. Mental health, emotional well-being, physical health, oral health, existential well-being and family QoL were all found to be significant domains associated with reductions in HRQoL in those who reported self-harm/suicidality.

Furthermore, evidence of the association between self-harm/suicidality and family QoL suggests that family might play a significant role for young people who are under 18 years old and have these thoughts and behaviours. This result was in line with previous research that emphasized the impacts of various family-related factors on self-harm/suicidality in this population. For example, there was evidence from two studies that those who engaged in self-harm reported impairments in general family functioning and had lower quality interaction with their parents than those without these behaviours (Klemera et al., 2017; Palmer et al., 2016). Another study found that young people from single-parent families were more likely to be chronic self-harmers compared to those from two-parent families (Burešová et al., 2015). These individuals may be exposed to excess social stress including discrimination, stigma and concealment (Botha and Frost, 2018). The fact is that family environment is crucial for the development of coping ability for people under 18 years of age and connections with parents are important for the maintenance of emotional well-being and health for young people, especially during adolescence (Bureau et al., 2010; Klemera et al., 2017). Factors such as parental ignorance, high parental behavioural control, overprotection, less warm emotional support or the absence of a family confidant may cause a young person to experience a lack of support to deal with their stressful life events, resulting in increasing likelihood of self-harm (Burešová et al., 2015; Fortune et al., 2016; Gyori et al., 2021; Rissanen et al., 2009; Tulloch et al., 1997). It should be noted that not all scales and instruments include family domains. Therefore, developmental trajectories should be considered when choosing appropriate HRQoL measurements for each age group in young populations. For example, family QoL domains might be more important to younger children while other domains such as work, romantic relationships and sexuality might be more important to young adults above 18 years of age (Titman et al., 1997).

Another important issue related to HRQoL measurement in young people is the issue of informants. Although many instruments rely substantially on child/adolescent self-report, self-report questionnaires may be problematic, particularly for younger children who lack the necessary language skills, cognitive capacity and long-term view of events (Solans et al., 2008; Theunissen et al., 1998). Consequently, parent/proxy reports might be a good substitute, although, as demonstrated, parents and children do not always agree in the completion of the questionnaires. This is not an uncommon finding; previous studies have shown that parents tend to rate their child’s HRQoL better in cases of nonclinical samples whereas parents tend to underestimate HRQoL of children with health conditions (Upton et al., 2008). Additionally, parents and children tend to agree more when assessing child behaviours such as physical functioning that are observable; but are less likely to do so in relation to emotion or social HRQoL behaviours, which are unobservable (Eiser and Morse, 2001). Furthermore, it should be noted that proxies might know less about a person’s suicidal thoughts and behaviours.

Findings suggest that the association between HRQoL and self-harm/suicidality could be in either direction or reciprocal. All of the studies included in this review were cross-sectional and none were longitudinal. Thus, there were no findings about how the relationship between HRQoL and self-harm/suicidality plays out over time and the direction of causality could not be inferred. In addition, findings on the association between HRQoL and self-harm/suicidality in relation to a mediational relationship with other factors implied that there might be other mechanisms driving their association. On the one hand, there might be aetiological factors that impact both HRQoL and self-harm/suicidality. For example, more emotional and peer problems resulted in lower HRQoL that, in turn, increased suicidal risk (Balazs et al., 2018). On the other hand, HRQoL may affect self-harm/suicidality indirectly through another factor. For example, environmental factors (presented by a low HRQoL outcome) might cause mental disorders or lead to hopelessness that correspondingly results in self-harm/suicidality (Gyori et al., 2021; Kwok and Shek, 2010). Elaborating these mechanisms underscores the value of measuring HRQoL in order to develop suicide prevention strategies, particularly in the case of interventions focusing on factors such as mental disorders or emotional and peer difficulties. Finally, findings from the group of people with suicidality showed that suicidal attempt and suicidal ideation were not related to HRQoL in the same way. The finding held true when considering both well-being and life satisfaction scales and non-preference-based HRQoL instruments. Therefore, a young person’s HRQoL may change possibly as the severity of suicidality increases (from ideation to attempt). An important issue to note is that the inconsistent findings concerning the association between HRQoL and suicidal ideation were mainly reported from moderate or weak studies with weak study designs and/or confounder components. Thus, further research based on longitudinal study designs with elaborated confounder analysis plans is strongly needed to fill these gaps in the literature.

The current review has some limitations. First, although a comprehensive search was conducted across many databases, the review excluded studies that were not published in English. Therefore, this may limit the relevance of our findings in non-English cultures and mean that we retrieved fewer studies from low- and middle-income countries. Second, it is possible that we might have missed studies where HRQoL was one of many exposure variables for self-harm/suicidality and was not explicitly listed in the title and abstract of the given article. Third, the diversity and heterogeneity of populations and scales/instruments considered in the studies presented a challenge for the review team in synthesizing results and drawing relevant conclusions. Finally, despite the importance of HRQoL as an outcome measurement for humans’ well-being and functioning in health research, it is undeniable that the construct of HRQoL has its own limitations. There has been an argument that HRQoL instruments mostly focus on a person’s functioning ability rather than well-being or how the person values his or her own health status (Guyatt, 1997). The argument was reinforced by specific examples of people with posttraumatic quadriplegia or having diagnoses of severe autism. Those individuals presented a poor HRQoL outcome as a result of limitations on several aspects of functioning but valued their life highly and profoundly, and were genuinely happy (Chapman and Carel, 2022; Guyatt, 1997). This lends support to the view that HRQoL may be conceptualized differently by different groups of people based on a variety of factors such as culture, ethnicity and neurodiversity (Chapman, 2021; Chapman and Carel, 2022). Despite many HRQoL instruments continuing to be developed, there are no universally accepted measurements across disciplines (Siette et al., 2021). These limitations remain a challenge for further research that is based on elaborating HRQoL in the context of suicide prevention.

## Conclusion

This review provided an overview of literature on the relationship between HRQoL and self-harm/suicidality in young people. Results from included studies showed there was an inverse association between self-harm/suicidal attempt and overall HRQoL irrespective of whether the included studies used well-being scales, life satisfaction scales, preference- or non-preference-based HRQoL instruments. Findings on specific QoL domains also suggested a number of QoL domains to be significantly associated with self-harm/suicidality in young people. We also identified different HRQoL according to the severity of suicidality (from ideation to attempt). Further research based on longitudinal study designs is required to fill these gaps in the literature.

## Supplemental Material

sj-docx-1-anp-10.1177_00048674231165477 – Supplemental material for Health-related quality of life in children, adolescents and young adults with self-harm or suicidality: A systematic review Click here for additional data file. Supplemental material, sj-docx-1-anp-10.1177_00048674231165477 for Health-related quality of life in children, adolescents and young adults with self-harm or suicidality: A systematic review by Ngoc Le, Yared Belete Belay, Long Khanh-Dao Le, Jane Pirkis and Cathrine Mihalopoulos in Australian & New Zealand Journal of Psychiatry

## Notes

1. In this paper, the phrase ‘young people’ will be used as an alternative to ‘children, adolescents and young adults’ to refer to people under 25 years of age.
