# Supplementary material for: Health-related quality of life in children, adolescents and young adults with self-harm or suicidality: A systematic review
Source: Aust N Z J Psychiatry. 2023 Apr 10;57(7):952–65. doi: 10.1177/00048674231165477 (PMC10291375; doi:10.1177/00048674231165477)
Supplement: sj-docx-1-anp-10.1177_00048674231165477 – Supplemental material for Health-related quality of life in children, adolescents and young adults with self-harm or suicidality: A systematic review [file sj-docx-1-anp-10.1177_00048674231165477.docx]

**APPENDIX 1**

**SEARCH TERMS**

Search terms were included using a combination of the following blocks:

| 1 | “Quality of Life” OR “QoL” OR “HRQoL” OR "health state utilit*" OR "utility score*" OR "utility value*" OR wellbeing OR “well-being” |
| --- | --- |
| AND | |
| 2 | “automutilat*” OR “overdose” OR “self-inflict*” OR “self-murder” OR “self-kill*” OR “self-destruct*” OR “self-mutilat*” OR “self-burn*” OR “self-cutting” OR “self-poisoning” OR “self-injur*” OR “self-harm” OR suicid* |
| AND | |
| 3 | Child* OR Toddler* OR adolescen* OR teen* OR “young people” OR “young person” OR “young adult*” OR youth* OR boy* OR girl* OR kid OR kids OR p#ediatr* OR juvenil* |

**APPENDIX 2**

**Quality assessment of the included studies**

| **Study** | **Selection bias** | **Study design** | **Confounders** | **Blinding** | **Data collection methods** | **Withdrawals and dropouts** | **Global rating** |
| --- | --- | --- | --- | --- | --- | --- | --- |
| Al-Bitar 2022 | Strong | Moderate | Weak | Moderate | Strong | Strong | Moderate |
| Algorta 2011 | Moderate | Moderate | Weak | Strong | Strong | Strong | Moderate |
| Balazs 2018 | Weak | Moderate | Strong | Moderate | Strong | Moderate | Moderate |
| Ballester 2021 | Strong | Moderate | Strong | Moderate | Strong | Strong | Strong |
| Díez-Gómez 2020 | Moderate | Moderate | Strong | Moderate | Strong | Moderate | Strong |
| Fonseca-Pedrero 2018 | Weak | Moderate | Weak | Moderate | Strong | Moderate | Weak |
| Gyori 2021 | Moderate | Moderate | Strong | Moderate | Strong | Moderate | Strong |
| Kwok 2010 | Moderate | Weak | Weak | Moderate | Strong | Moderate | Weak |
| Le 2021 | Strong | Moderate | Strong | Moderate | Strong | Moderate | Strong |
| Lee 2017 | Strong | Weak | Strong | Moderate | Strong | Moderate | Moderate |
| Luo 2021 | Strong | Moderate | Strong | Moderate | Strong | Weak | Moderate |
| Morey 2017 | Weak | Moderate | Weak | Moderate | Strong | Moderate | Weak |
| Owusu-Ansah 2020 | Moderate | Weak | Weak | Moderate | Strong | Moderate | Weak |
| Parker 2018 | Strong | Moderate | Weak | Moderate | Moderate | Strong | Moderate |
| Quarshie 2021 | Strong | Weak | Strong | Moderate | Moderate | Strong | Moderate |
| Resch 2008 | Moderate | Moderate | Moderate | Moderate | Strong | Moderate | Strong |
| Rönkä 2013 | Strong | Moderate | Strong | Moderate | Strong | Moderate | Strong |
| Sisask 2008 | Weak | Weak | Strong | Moderate | Strong | Moderate | Weak |
| Taliaferro 2009 | Strong | Weak | Strong | Moderate | Strong | Moderate | Moderate |
| Thatcher 2002 | Strong | Moderate | Strong | Moderate | Strong | Strong | Strong |
| Wang 2019 | Strong | Moderate | Strong | Moderate | Strong | Strong | Strong |
| Whitlock 2015 | Weak | Weak | Strong | Moderate | Strong | Moderate | Weak |
| Zullig 2016 | Weak | Moderate | Strong | Moderate | Strong | Moderate | Moderate |
|  |  |  |  |  |  |  |  |
